# Comparison of Proteomic and Transcriptomic Profiles in the Bronchial Airway Epithelium of Current and Never Smokers

**DOI:** 10.1371/journal.pone.0005043

**Published:** 2009-04-09

**Authors:** Katrina Steiling, Aran Y. Kadar, Agnes Bergerat, James Flanigon, Sriram Sridhar, Vishal Shah, Q. Rushdy Ahmad, Jerome S. Brody, Marc E. Lenburg, Martin Steffen, Avrum Spira

**Affiliations:** 1 The Pulmonary Center, Boston University Medical Center, Boston, Massachusetts, United States of America; 2 Bioinformatics Program, College of Engineering, Boston University, Boston, Massachusetts, United States of America; 3 Newton-Wellesley Hospital, Newton, Massachusetts, United States of America; 4 Department of Pathology and Laboratory Medicine, Boston University School of Medicine, Boston, Massachusetts, United States of America; 5 The Broad Institute of Harvard and MIT, Cambridge, Massachusetts, United States of America; University of Pittsburgh, United States of America

## Abstract

**Background:**

Although prior studies have demonstrated a smoking-induced field of molecular injury throughout the lung and airway, the impact of smoking on the airway epithelial proteome and its relationship to smoking-related changes in the airway transcriptome are unclear.

**Methodology/Principal Findings:**

Airway epithelial cells were obtained from never (n = 5) and current (n = 5) smokers by brushing the mainstem bronchus. Proteins were separated by one dimensional polyacrylamide gel electrophoresis (1D-PAGE). After in-gel digestion, tryptic peptides were processed via liquid chromatography/ tandem mass spectrometry (LC-MS/MS) and proteins identified. RNA from the same samples was hybridized to HG-U133A microarrays. Protein detection was compared to RNA expression in the current study and a previously published airway dataset. The functional properties of many of the 197 proteins detected in a majority of never smokers were similar to those observed in the never smoker airway transcriptome. LC-MS/MS identified 23 proteins that differed between never and current smokers. Western blotting confirmed the smoking-related changes of PLUNC, P4HB1, and uteroglobin protein levels. Many of the proteins differentially detected between never and current smokers were also altered at the level of gene expression in this cohort and the prior airway transcriptome study. There was a strong association between protein detection and expression of its corresponding transcript within the same sample, with 86% of the proteins detected by LC-MS/MS having a detectable corresponding probeset by microarray in the same sample. Forty-one proteins identified by LC-MS/MS lacked detectable expression of a corresponding transcript and were detected in ≤5% of airway samples from a previously published dataset.

**Conclusions/Significance:**

1D-PAGE coupled with LC-MS/MS effectively profiled the airway epithelium proteome and identified proteins expressed at different levels as a result of cigarette smoke exposure. While there was a strong correlation between protein and transcript detection within the same sample, we also identified proteins whose corresponding transcripts were not detected by microarray. This noninvasive approach to proteomic profiling of airway epithelium may provide additional insights into the field of injury induced by tobacco exposure.

## Introduction

Cigarette smoking, the leading cause of preventable death in the United States, is responsible for 440,000 deaths per year[1], [2]. Smoking is the single most important risk factor in the development of lung cancer, the leading cause of cancer related death in the U.S., and of chronic obstructive pulmonary disease (COPD), the fourth leading cause of death overall[2]. Although smoking is strongly associated with diseases such as lung cancer and COPD, the mechanisms by which smoking contributes to their pathogenesis are not completely understood.

Cigarette smoke creates a field of molecular injury in the epithelial cells lining the entire respiratory tract. Changes include cellular atypia[3], allelic loss[4]–[6], and promoter hypermethylation[7]. Using oligonucleotide arrays and candidate gene approaches, our group and others have previously identified a number of mRNA expression changes that occur in the histologically normal airway epithelium in response to smoking[8]–[12] and in association with disease[13]–[16]. Furthermore, we have recently described smoking-induced changes in airway microRNA expression and their potential role in regulating the mRNA response to tobacco smoke [17]. In this study, we sought to extend this field of molecular injury to the protein level and characterize the effect of smoking on the airway epithelium proteome.

Prior studies have analyzed lung tissue from never, current and former smokers using two-dimensional electrophoresis (2DE) coupled with mass spectrometry, leading to the hypothesis that smoke exposure induces an unfolded-protein-like response [18]. Other studies identified lung-cancer-specific proteomic differences in bronchial epithelium obtained by biopsy from both “healthy” smokers and smokers with a history of lung cancer[19], [20]. Though studies have been performed using pooled nasal lavage samples[21] and pooled exhaled breath condensate samples[22], little is known about either the effects of smoking on the proteome of airway epithelial cells, or the variability in this response between individuals. In the current study we examined the effects of smoking on the airway epithelial proteome by analyzing individual samples collected by bronchoscopy from the mainstem bronchus. The ability to collect data from individual samples lays the ground work for understanding variation in the proteomic response to cigarette smoke between individuals which may ultimately be useful for determining why only a subset of smokers develop lung cancer or COPD.

Although studies have tried to address the large-scale correlation between protein production and mRNA expression in both cell lines[23]–[39] and human tissues[40]–[46], the findings have been variable. Studies of yeast and human liver tissue have yielded moderate correlation of protein abundance to mRNA expression[23], [36]–[38], [43]. A strong correlation has been reported for abundant proteins in an epithelial cell line model of ErbB-2 overproduction in breast cancer[39]; however, protein abundance and levels of mRNA expression have correlated poorly in resected lung adenocarcinomas[45], [46]. The relationship between protein production and mRNA expression in normal airway epithelium remains unclear, as does the impact of smoking on this relationship.

In this study, we profiled proteins and genes expressed within the same bronchial epithelium of never and current smokers via 1D-PAGE with LC-MS/MS and DNA microarrays respectively. The relationship between protein detection and mRNA expression was explored both globally and for individual proteins of interest. We found that the majority of airway proteins detected by mass spectrometry have their corresponding transcripts detected at measurable levels by microarray, and that changes at the protein level in response to cigarette smoke parallel smoking-induced changes in mRNA. This approach also detected proteins whose corresponding transcript expression was not detected by microarrays. This study represents the first application of this approach to the simultaneous proteomic and transcriptomic profiling of airway epithelium within the same individual, providing insight into the normal and smoking-affected airway proteome and the relationship between protein changes and the previously described changes in airway gene expression.

## Results

### Study Population

The idemographics for subjects recruited into this study are shown in Table 1. The never and current smokers differed in age and cumulative tobacco exposure (as measured by pack-years of smoking) (p<0.05), but were similar for other demographics. None of the subjects were using inhaled medications.

**Table 1:** Demographics of the 10 subjects undergoing bronchoscopy.

*Sample*	*Age*	*Sex*	*Cumulative Tobacco Exposure (Pack Years)*	*FVC%*	*FEV_1_%*	*FEV_1_/FVC*
NS1	23	Male	0	101%	96%	0.82
NS2	32	Male	0	88%	97%	0.91
NS3	28	Male	0	98%	101%	0.87
NS4	32	Female	0	108%	111%	0.89
NS5	27	Male	0	127%	140%	0.92
CS1	34	Male	17	87%	84%	0.81
CS2	34	Female	15	84%	85%	0.72
CS3	45	Female	14	90%	94%	0.88
CS4	45	Male	16	88%	97%	0.91
CS5	47	Male	39.5	91%	89%	0.81

NS indicates never smokers, and CS indicates current smokers. FVC indicates the forced vital capacity as a percent of the predicted value. FEV_1_% indicates the forced expiratory volume at one second as a percent of the predicted value. A Student's t-test was performed for continuous variables, and a chi square test for dichotomous variables. Never and current smokers differed in age and pack years of smoking (p<0.05).

### Normal Airway Proteome

**Figure 1:**
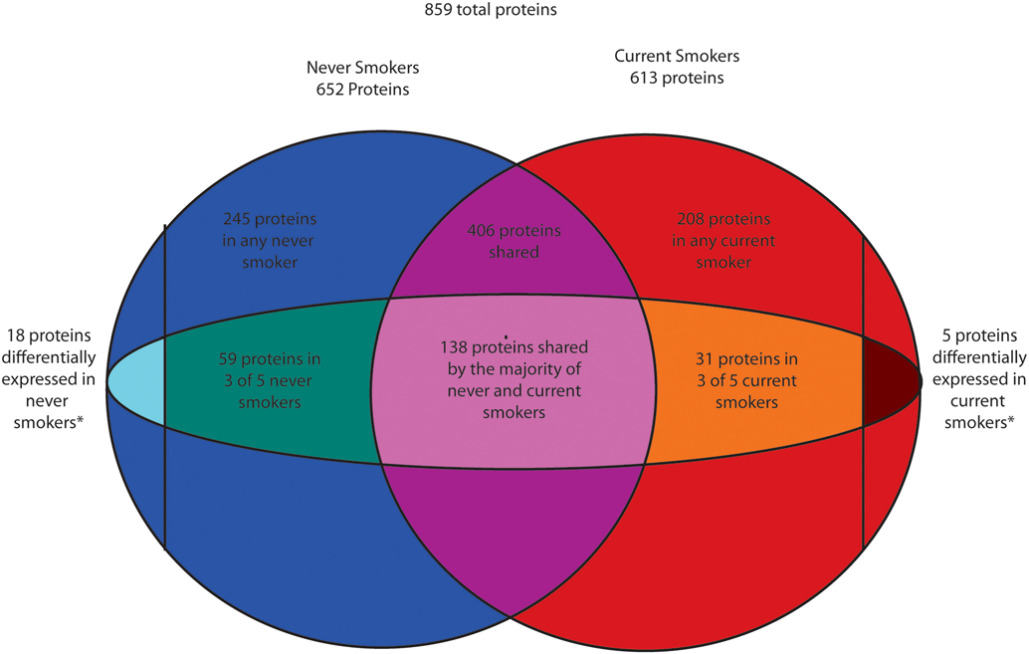
Venn diagram describing the proportion of proteins detected in never and current smokers. The circles represent proteins detected in at least one sample. A total of 859 proteins were detected by LC-MS/MS in any sample. 652 proteins were detected by LC-MS/MS in any never smoker, and 613 proteins were detected in at least one current smoker. The inner oval represents proteins detected by LC-MS/MS in the majority of samples. 197 proteins were detected in the majority of never smokers, and 169 proteins were detected in the majority of current smokers. *A total of 23 proteins differ between never and current smokers based on the criteria described in the methods.

A total of 652 proteins were detected in one or more never smokers, with 197 proteins found in the majority of never smokers (Figure 1). Proteins with molecular functions related to airway biology were over-represented among this list (Table 2). The functional categorization of the normal airway proteome was compared to over-represented functional categories of the normal airway transcriptome among transcripts detected by microarray both in these same five never smoker samples as well as a larger previously described cohort of 22 never smokers [8]. mRNAs and proteins associated with nucleotide binding, and pyrophosphate activity were over-represented in both datasets (P_DAVID-BH_<0.05).

**Table 2:** Enriched functions in the never smoker airway proteome.

*Molecular Functions*	*P-Value*	*FDR*
**Binding**		
***Nucleotide binding***	8.9*10^−5^	3.5*10^−2^
**Catalytic activity**		
***Hydrolase activity, acting on acid anhydrides***	1.0*10^−5^	7.0*10^−3^
***Hydrolase activity, acting on acid anhydrides, in phosphorous-containing anhydrides***	9.6*10^−6^	8.8*10^−3^
***Pyrophosphatase activity***	8.5*10^−6^	1.2*10^−2^
**Nucleoside-triphosphate activity**	8.4*10^−6^	2.3*10^−2^
***Oxidoreductase activity***		
Oxidoreductase activity, acting on the Aldehyde or oxo Group of donors	7.0*10^−5^	3.2*10^−2^
Oxidoreductase activity, acting on the Aldehyde or oxo group of donors, NAD or NADP as acceptor	3.3*10^−5^	1.8*10^−2^

Statistically enriched functional categories (FDR<0.05) and subcategories of the 197 proteins detected in the majority of never smokers as determined by DAVID. Over-represented categories that contain more than two probe sets are included. Functional categories that are also over-represented (FDR<0.05) among transcripts detected in the all never smokers in this cohort are **bolded**. Functional categories that are also enriched (FDR<0.05) among transcripts detected in all never smokers from a previously published cohort [8] are *italicized*.

### Effect of Cigarette Smoking on the Large Airway Proteome

613 proteins were detected in one or more current smokers, and 169 proteins were detected in the majority of current smokers (Figure 1). Three proteins differed in their rate of detection between current and never smokers at P_Fisher_≤0.05. Aldehyde dehydrogenase 3B1 (ALDH3B1, NP_000685), a gene highly expressed in lung[47], was detected in all five never smokers and only one current smoker (P_Fisher_ = 0.048). Palate, lung and nasal epithelium carcinoma associated protein precursor (PLUNC, NP_570913), a secretory protein in the upper respiratory tract was detected in four never smokers and absent in all current smokers (P_Fisher_ = 0.048). Hypothetical protein DKFZP586A0522 protein (NP_054752) was also detected in four never smokers and absent in all current smokers (P_Fisher_ = 0.048).

Due to the small sample size, a second list of differentially detected proteins was defined using a qualitative criterion: proteins were included if present in three or more samples of one class compared to the other. Twenty-three proteins differed between never and current smokers based on these criteria (Table 3).

**Table 3:** Proteins differentially detected in the airway of never and current smokers by mass spectrometry.

*Protein Name*	*RefSeqID*	*#Nevers / #Currents*
alpha-2-macroglobulin precursor	NP_000005	0/3
transferrin; PRO2086 protein	NP_001054	0/3
ribosomal protein S2; 40S ribosomal protein S2	NP_002943	1/4
superoxide dismutase 2, mitochondrial	NP_000627	2/5
prolyl 4-hydroxylase, beta subunit	NP_000909	2/5
S-adenosylhomocysteine hydrolase	NP_000678	3/0
aldehyde dehydrogenase 9A1	NP_000687	3/0
dynein, axonemal, heavy polypeptide 5	NP_001360	3/0
dynein, axonemal, heavy polypeptide 9 isoform 2	NP_001363	3/0
dynein, cytoplasmic, heavy polypeptide 1	NP_001367	3/0
prostatic binding protein	NP_002558	3/0
phosphoglycerate mutase 1 (brain)	NP_002620	3/0
secretoglobin, family 1A, member 1 (uteroglobin)	NP_003348	3/0
Fc fragment of IgG binding protein	NP_003881	3/0
aminopeptidase puromycin sensitive	NP_006301	3/0
arachidonate 15-lipoxygenase	NP_001131	4/1
S100 calcium binding protein A11	NP_005611	4/1
valosin-containing protein	NP_009057	4/1
**DKFZP586A0522 protein**	**NP_054752**	**4/0**
**palate, lung and nasal epithelium carcinoma associated protein precursor**	**NP_570913**	**4/0**
CGI-38 protein	NP_057048	5/2
tubulin beta MGC4083	NP_115914	5/2
**aldehyde dehydrogenase 3B1**	**NP_000685**	**5/1**

The proteins that are differentially detected in never and current smokers are listed by protein name and by RefSeq identification number. The right column shows the numbers of never and current smokers samples in which the protein was detected. Proteins with a Fisher exact p≤0.05 comparing never and current smokers are shown in **bold**.

### Western Blotting

**Figure 2:**
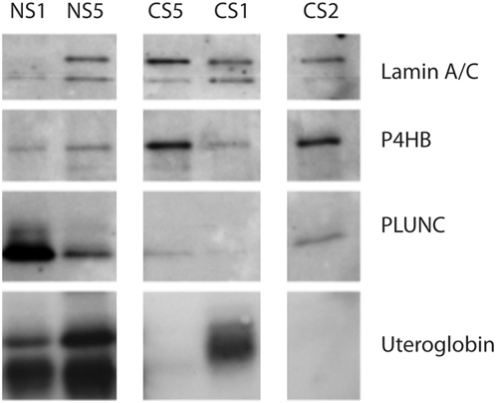
Western blot validation of proteins detected by proteomics in never and current smokers. Western blotting shows significantly higher levels of PLUNC in the never smokers. Higher levels of uteroglobin were also observed in never smokers, although there was heterogeneity among the current smokers. There was a small increase in P4HB in two of the current smoker samples.

We validated mass spectrometry findings by immunoblot for three of the proteins that differed between never and current smokers (Figure 2). PLUNC, uteroglobin and P4HB were selected from the list of twenty-three candidates based on their biologic interest, molecular weight, and antibody availability. Of these, PLUNC also had a Fisher exact p-value<0.05. Decreased levels of PLUNC and uteroglobin were confirmed among current smokers, although there was heterogeneity for uteroglobin among current smokers (Figure 2). P4HB levels were elevated in two of the current smokers as compared to two never smokers.

### Comparison of Protein and mRNA Expression

An average of 93% of proteins detected by mass spectrometry had at least one matching probe set on the HG-U133A array. Of these, an average of 86% had detectable gene expression (P_detection_<0.05) in samples collected from the same participants demonstrating a significant level of co-detection (χ^2^ = 347, p = 2.2×10^−16^). There was not a significant difference in the rate of co-detection between never and current smokers.

**Figure 3:**
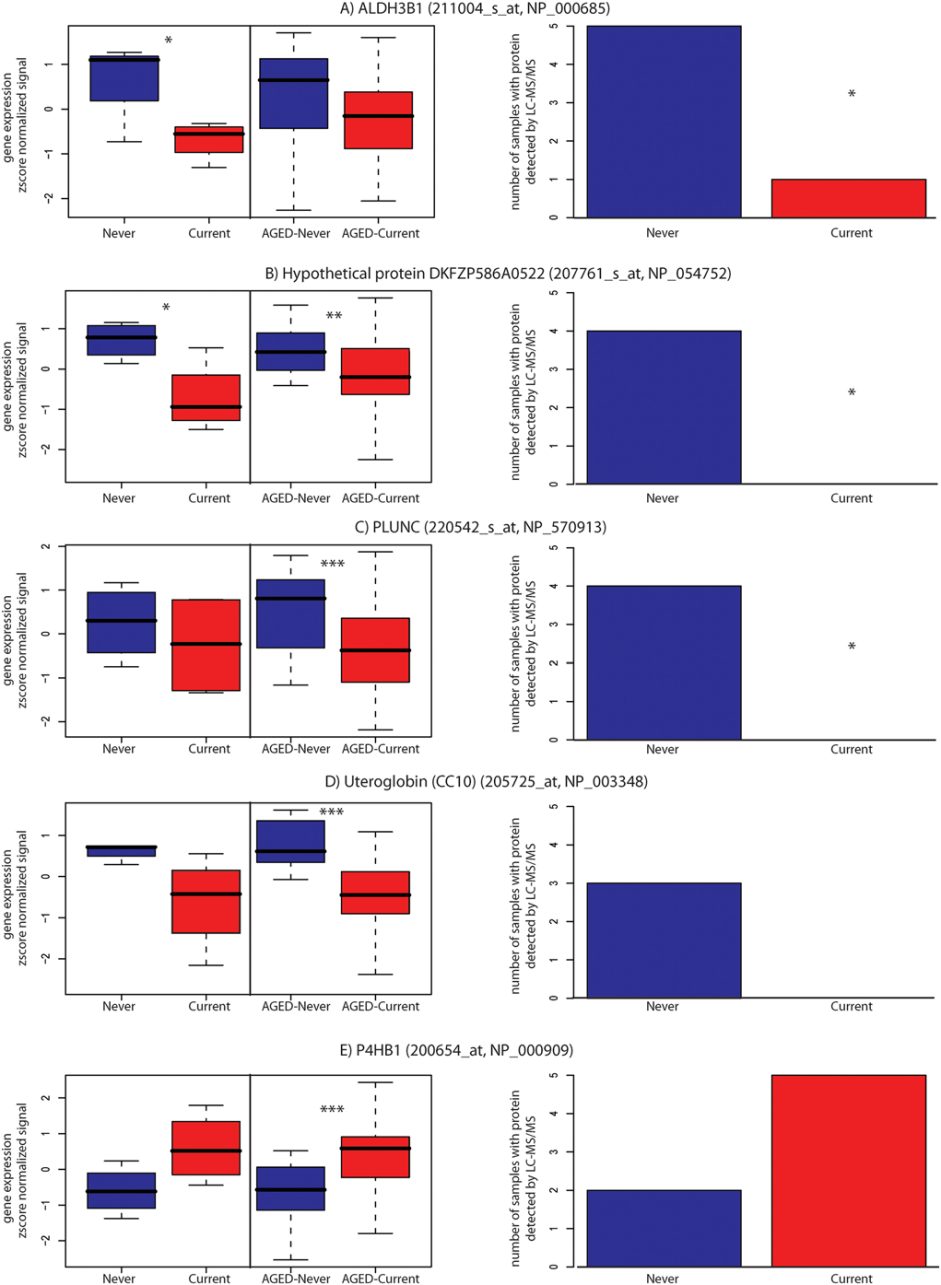
Comparison of individual protein detection and mRNA expression. Boxplots of the gene expression levels and bar graphs of LC-MS/MS results for A) ALDH3B1, B) hypothetical protein DKFZP586A0522, C) PLUNC, D) uteroglobin (CC10), and E) P4HB subunit. The borders of each boxplot represent the interquartile range of z-score normalized natural logarithm of the MAS5 gene expression data from this cohort of 5 never smoker and 5 current smokers, and from a previously published cohort (AGED) of 23 never smokers and 34 current smokers, excluding one never smoker in common to this study. The solid line within each box represents the median gene expression, and the whiskers of the plot extend to the upper and lower extremes of the data for each gene. Bar plots represent the number of smoker and nonsmoker samples in the current study where the protein was detected. Proteomic analysis detected ALDH3B1, hypothetical protein DKFZP586A0522, PLUNC and uteroglobin in more never smokers, while P4HB was detected in more current smokers. There is concordance in the direction of change for smoking-related protein and gene expression changes for these 5 genes. * p<0.05. ** p<0.005. *** p<0.0005.

For select proteins where detection varied between never and current smokers, we examined the expression of the corresponding mRNA for smoking-related differential expression. PLUNC (NP_570913), ALDH3B1 (NP_000685), and hypothetical protein DKFZP586A0522 (NP_054752) were selected based on the results of the Fisher exact test. Uteroglobin (NP_003348) and the prolyl 4-hydroxylase beta subunit (P4HB) (NP_000909) were selected based on their qualitative differences between never and current smokers. Within this cohort, mRNA expression positively correlated with protein detection for PLUNC, uteroglobin, and P4HB (Figure 3).

The association between smoking and gene expression was also examined in a previously published cohort [8] from which we excluded a sample that overlapped with the samples used in this study (Figure 3). Consistent with the protein detection data and the gene expression data from the present study, in this independent group of never and current smokers, *ALDH3B1*, *hypothetical protein DKFZP586A0522*, *PLUNC* and *uteroglobin* mRNA expression were higher in never smokers and *P4HB* gene expression was higher in current smokers. Additionally, we used this cohort to assess the potential confounding effects of age on the smoking-induced changes in candidate proteins identified in the current study. Within the previously published cohort, we identified 12 never and 12 current smokers matched within 1 year for age. A t-test performed on these age-matched 12 never smokers and 12 current smokers confirmed differential gene expression of *ALDH3B1* (211004_s_at, p = 0.03), *hypothetical protein DKFZP586A0522* (207761_s_at, p = 0.03), *PLUNC* (220542_s_at, p = 0.02), *uteroglobin* (205725_at, p = 0.0005), and *P4HB1* (200654_at, p = 0.03).

Differences in protein detection by mass spectrometry and transcript detection by microarray were also explored. In the matched samples, there was no expression by microarray of transcripts corresponding to 41 proteins that were detected in ≥50% of samples by mass spectrometry (Table 4). Additionally, expression of these transcripts was detected in ≤5% of the never and current smokers in the larger previously published dataset[8] of never and current smokers. Ten of these 41 proteins have been previously described in the erythrocyte proteome[48], which is not surprising given that brushings contain small numbers of red blood cells that lack nucleic acids.

**Table 4:** Proteins detected in the airway by mass spectrometry that lack detectable transcript by microarray.

*Protein Name (RefSeqID)*	
Actin, alpha 1, skeletal muscle (NP_001091)^1^^,^ 3	Succinate dehydrogenase complex, subunit B, iron sulfur (Ip) (NP_002991)^5^
Myosin, heavy polypeptide 14 (NP_079005)^1^^,^ 3	Superoxide dismutase 2, mitochondrial (NP_000627)^5^
Tubulin, beta 1 (NP_110400)^1^^,^ 3	Phosphorylase, glycogen; brain (NP_002853)^6^
Tubulin, beta 4 (NP_006078)^1^^,^ 3	Phosphorylase, glycogen; muscle (McArdle syndrome, glycogen storage disease type V) (NP_005600)^6^
Spectrin, alpha, non-erythrocytic 1 (alpha fodrin) (NP_003118)^1^^,^ 2 ^,^ 3 ^,^ 4	3-hydroxyisobutyrate dehydrogenase (NP_689953)^8^
Spectrin, beta, non-erythrocytic 1 (NP_842565)^1^^,^ 2 ^,^ 3 ^,^ 4	Adenylate kinase 1 (NP_000467)^8^^,^ 9
Villin 2 (ezrin) (NP_003370)^1^^,^ 2 ^,^ 3 ^,^ 4	N-acylsphingosine amidohydrolase (acid ceramidase) 1 (NP_808592)^8^
Histone 1, H1b (NP_005313)^1^	Apolipoprotein A-I (NP_000030)^8^
Histone 1, H3f (NP_066298)^1^	Cytochrome c oxidase subunit IV isoform 1 (NP_001852)^8^
Histone 1, H4k (NP_068803)^1^	Heat shock 70 kDa protein 1-like (NP_005518)^8^
RAB6A, member RAS oncogene family (NP_002860)^1^	Heat shock 70 kDa protein 6 (HSP70B′) (NP_002146)^8^
Albumin (NP_000468)^1^^,^ 5 ^,^ 6 ^,^ 7	Heterogeneous nuclear ribonucleoprotein C (C1/C2) (NP_112604)^8^
Karyopherin (importin) beta 1 (NP_002256)^1^^,^ 5	Heterogeneous nuclear ribonucleoprotein M (NP_005959)^8^
Lamin A/C (NP_733821)^3^	Peptidylprolyl isomerase A (cyclophilin A) (NP_066953)^8^^,^ 9
Lamin B2 (NP_116126)^3^	Peroxiredoxin 2 (NP_005800)^8^^,^ 9
Stomatin (NP_004090)^3^^,^ 9	Phosphoglycerate kinase 1 (NP_000282)^8^
Carbonic anhydrase I (NP_001729)^5^^,^ 9	Pyruvate kinase, muscle (NP_002645)^8^
Carbonic anhydrase II (NP_000058)^5^^,^ 9	Solute carrier family 4, anion exchanger, member 1 (erythrocyte membrane protein band 3, Diego blood group) (NP_000033)^8^
Catalase (NP_001743)^5^^,^ 9	Tumor rejection antigen (gp6) 1 (NP_003290)^8^
Hemoglobin, delta (NP_000510)^5^^,^ 7 ^,^ 9	Voltage-dependent anion channel 3 (NP_005653)^8^
Hemoglobin, gamma A (NP_000550)^5^^,^ 7 ^,^ 9	

A total of 41 proteins detected in at least half of the samples by LC/MS-MS lacked detectable expression by microarray at a detection p-value<0.05. Fewer than 5% of airway samples from a previously published dataset[8] had detectable expression of a transcript corresponding to these proteins.

1 Cell organization and biosynthesis (P_DAVID_<0.05).

2 Cortical cytoskeleton (P_DAVID_<0.05).

3 Cytoskeleton (P_DAVID_<0.05).

4 Cell cortex (P_DAVID_<0.05).

5 Transition metal ion binding (P_DAVID_<0.05).

6 Pyridoxal phosphate binding (P_DAVID_<0.05).

7 Oxygen binding (P_DAVID_<0.05).

8 Unclassified in DAVID.

9 Component of the erythrocyte proteome [48].

## Discussion

We applied 1D-PAGE coupled with LC-MS/MS to the study of the airway epithelium proteome and its response to cigarette smoke exposure. This study presents the first proteomic profile of a relatively pure population of bronchial epithelial cells obtained from bronchoscopy brushings. We also used differences in the rate of protein detection between never and current smokers to identify candidates for proteins that vary in abundance in response to tobacco-smoke exposure. The effect of smoking on several of these proteins was confirmed by Western blot. We also found that for many candidates, smoking similarly affected expression of the mRNA transcripts that gave rise to these proteins. This was accomplished by measuring gene expression in the same samples that were profiled at the proteomic level and in an independent data set. The majority of proteins identified by LC-MS/MS had detectable levels of their corresponding transcript by microarray. Differing methodologies may account for the stronger relationship between protein and gene expression reported here relative to prior studies[36], [39], [43], [45], [46].

Analysis of the proteome using 1D-PAGE coupled with LC-MS/MS resulted in the detection of 41 proteins for which expression of corresponding transcripts was not detected by microarray. Some of these failures to detect transcript expression could represent technical limitations of the microarray platform. However, we were intrigued that several of the proteins whose transcripts were not detected by microarray represent erythrocyte-specific proteins. This suggests that: 1) the airway epithelial samples collected for this study were likely contaminated with erythrocytes, and 2) that more generally, stable proteins may be detected by proteomic methods long after the mRNA which encodes for them has disappeared.

Using habitual smoking as a paradigm for inhalational exposures affecting airway epithelium, we have identified changes in protein among smokers by LC-MS/MS and validated select changes with Western blotting. A decrease in the short isoform of PLUNC has previously been described in the pooled nasal lavage fluid of current smokers when compared with nonsmokers[21]. Although the exact function of this protein is unclear, it is thought to act in the inflammatory response to inhaled irritants such as tobacco smoke. Other studies have demonstrated decreased levels of uteroglobin, an anti-inflammatory protein secreted by Clara cells, in the BAL[49], pooled nasal lavage fluid[21], and serum[50] of healthy smokers and in the bronchial epithelium of former smokers with COPD undergoing lung transplantation[51]. P4HB has been detected in a proteomic analysis of cell surface proteins of a lung adenocarcinoma cell line[52] and in the 2DE-proteomic analysis of resected lung adenocarcinomas[46]. This protein may function in the anti-oxidant response to cigarette smoke[46]. Other proteins with oxidoreductase activity identified by this approach, such as ALDH3B1, have not previously been linked to cigarette smoking at the protein level but may function in the airway epithelial response to the toxins in cigarette smoke. None of the proteins differentially detected in smokers in this study overlapped with proteins previously described as differentially expressed in the lungs of Winstar rats exposed to cigarette smoke[53], or proteins differentially detected by 2DE/MALDI-TOF in a human pneumocyte cell line exposed to cigarette smoke extract[54].

This study was limited by a relatively small sample size, the sensitivity of the proteomic technique, and challenges in the quantification of proteins. While age was a confounding variable in this study, the gene expression changes in the airway epithelium of never and current smokers were validated using age-matched samples from current and never smokers in a previously published gene-expression study [8], suggesting that the association between smoking-status and both gene and protein expression is unlikely to be due to differences in patient age. The amount of time elapsed between last smoking a cigarette and bronchoscopy was not recorded, and some of the variability of protein levels in Western blotting might relate to potential differences to the acute versus chronic effects of cigarette smoke. Although the small sample size limited the statistical analysis, Western blotting validated differences in protein detection identified by LC-MS/MS suggesting the method's potential specificity. However, the power of our study to detect additional proteomic changes that occurred in response to cigarette smoke exposure was limited. The sensitivity of this technology allowed detection of 859 proteins with a false positive rate of 1%. While this represents a small percentage of the total proteins present in epithelial cells, we have identified a greater number of proteins than previously used methods of sample collection and proteomic analysis for smokers and nonsmokers[20]–[22]. Because of the uncertainties associated with label-free quantification methods for the determination of protein expression levels, this platform serves mainly as a discovery tool. However, promising efforts in this area, including correlation of peak intensity or spectral counts with protein abundance, may soon remove this limitation[55]–[58].

In summary, we have described the proteomic profile of normal bronchial epithelial cells using 1D-PAGE coupled with LC-MS/MS and linked this profile to smoking-induced transcriptional changes in these same cells. This approach has the potential to provide additional insight into host response to tobacco smoke and the pathogenesis of smoking-related lung disease.

## Materials and Methods

### Study population, sample collection, and ethics statement

Never (n = 5) and current smokers (n = 5) were recruited for fiberoptic bronchoscopy at Boston Medical Center. Detailed medical and smoking histories were obtained including number of cigarettes smoked per day, cumulative tobacco exposure measured in pack-years, and an estimation of second-hand smoke exposure. Screening prior to bronchoscopy included an electrocardiogram, chest radiograph and spirometry. Participants with a history of underlying lung disease, significant second hand smoke exposure, an abnormal baseline EKG, or evidence of obstructive lung disease on spirometry (defined as an FEV_1_/FVC<0.7) were excluded from the study. This study was approved by the Institutional Review Board at Boston Medical Center, and all subjects provided written informed consent.

Bronchial epithelial cell brushings from the right mainstem bronchus were obtained at the time of bronchoscopy with an endoscopic cytology brush (Cellebrity Endoscopic Cytology Brush, Boston Scientific, Natick, MA). Cytokeratin staining has demonstrated that this method results in the collection of greater than 90% pure population of bronchial epithelial cells[8]. Airway brushings obtained for proteomics were immediately placed in PBS (Invitrogen, Carlsbad, CA). Additional brushes were collected for gene expression profiling and stored in TRIzol (Invitrogen). Samples in PBS were pelletted at 3500 rpm for 3 minutes, washed with PBS, and stored at −80°C until processing for mass spectrometry. The airway brushings in TRIzol were stored at −80°C until processing.

### Proteomic Sample Processing and Mass Spectrometry

After cell lysis with 2% SDS, proteins were separated on a 4–20% polyacrylamide minigel by electrophoresis and stained with Coomassie Blue (Supporting Figure S1). Each gel lane was cut into 35–70 sections. Proteins were reduced with DTT, alkylated with iodoacetamide, and digested with trypsin using a DigestPro 96 robot (Intavis Bioanalytical Instruments, Cologne, Germany). Extracted peptides were dried and resuspended in 0.5% acetic acid in preparation for mass spectrometry.

All samples were analyzed by LC-MS/MS using an LTQ ProteomeX ion trap mass spectrometer (ThermoFinnigan, Waltham, MA). Peptides from each gel slice were serially injected onto a home-packed C18 reverse-phase column (Magic C18AQ, 15 cm×100 micron ID, Michrom Bioresources, Inc., Auburn, CA) interfaced directly to the mass spectrometer. Peptides were separated using short, biphasic, 20-minute gradients of 0–90% acetonitrile in the presence of 0.5% acetic acid. From each parent ion scan (MS scan), the ten most intense ions were selected for collision-induced dissociation, and the spectra of the peptide fragments were recorded (MS2 scan).

### Protein Identification and Analysis

The data were analyzed using SEQUEST software[59]. Spectra were queried against the curated entries of the NCBI RefSeq database and Xcorr values adjusted for an empiric false positive identification rate of 1% for forward-sequence proteins as determined by the inclusion of reversed protein sequences[60]. Positive identification of a protein required observation of at least two matching peptides from the same or adjacent gel slices.

### Western Blotting

Residual protein lysates from two never and five current smoker samples were quantified by 1D-PAGE and Coomassie blue staining (Supporting Figure S2). Of these samples, sufficient material was available for Western blotting of two never smoker samples and four current smoker samples. One current smoker sample was excluded due to lack of signal from the loading control, lamin A/C. Samples were incubated at 86°C in SDS-sample buffer and electrophoresed on a 4–20% SDS-PAGE gel. Proteins were transferred to nitrocellulose and stained with Ponceau Red. The membrane was blocked with 5% nonfat milk in TBS-Tween and incubated with the appropriate primary and secondary antibodies. Mouse anti-human prolyl 4-hydroxylase beta subunit was obtained from Chemicon (Temecula, CA). Mouse anti-human PLUNC and goat anti-mouse-HRP affinity purified antibodies were purchased from R&D Systems (Minneapolis, MN). Rabbit anti-uteroglobin was obtained from Abcam (Cambridge, MA). Lamin A/C, a nuclear matrix protein, was used as a loading control.

### Microarray Sample Processing

Six to eight micrograms of RNA obtained from five of the never smoker and four of the current smoker participants was processed and hybridized to an Affymetrix HG-U133A GeneChip (Affymetrix Inc., Santa Clara, CA) containing ∼22,215 probesets as previously described[8].

### Microarray Data Acquisition and Preprocessing

Expression Console Version 1.0 (Affymetrix Inc.) was used to generate a MAS5 weighted-mean expression level for each transcript and a detection p-value (P_detection_), which indicates the reliability of detection of that transcript above background on the array. The mean intensity for each array was scaled to 100. Each array included in the final analysis had at least 30% of the probesets detected above background (percent present >30%) and a 3′ to 5′ ratio of signal intensity for GAPDH of less than or equal to 5. One never smoker microarray was excluded based on these quality control filters (low percent present, high 3′ to 5′ GAPDH ratio), leaving four never and four current smoker arrays for analysis.

Sample contamination with significant numbers of non-epithelial cells was evaluated, as described previously[8], by analyzing arrays for the presence of transcripts known to be present in airway epithelium and by confirming the absence of transcripts specific to non-epithelial cell types. No arrays were excluded based on these criteria.

### Comparison of Protein Detection and mRNA Expression

For each protein, we queried the microarray data from the same patient for expression (P_detection_<0.05) of a matching transcript. The significance of the overlap between detected proteins and transcripts was determined using Pearson's Chi-squared test with Yates' continuity correction.

A comparison of protein detection and transcript expression level was also performed for individual proteins of interest using the microarray data generated in this study and a previously published cohort of 23 never smokers and 34 current smokers [8], excluding one never smoker in common to this cohort. The transcript expression data for these samples was obtained from http://pulm.bumc.bu.edu/aged and log normalized. The association between smoking status and gene expression was determined as previously described [8].

### Functional Enrichment Analysis

Functional enrichment analysis was performed using DAVID (http://david.abcc.ncifcrf.gov/)[61]. A modified Fisher exact test (P_DAVID_) was calculated for all analyses, and the Benjamini-Hochberg method was used to correct for false discovery (P_DAVID-BH_). To determine the molecular functions that were over-represented within the never smoker proteome, the Gene Ontology (GO) molecular functions of the U133A probes corresponding to the proteins detected in the majority of never smokers were compared to the GO molecular functions of all probe sets on the U133A array. A similar analysis was also performed for the never smoker transcriptome. Genes expressed at P_detection_<0.05 in all never smokers with good quality microarrays were compared to a background of all genes represented by probe sets on the U133A microarray. A parallel analysis was performed in DAVID using the genes expressed at P_detection_<0.05 in the 22 unique never smokers from a previously published data set[8]. Over-represented gene ontology categories for proteins changed by smoking and for proteins that were not detectably expressed by microarray were determined by comparing the corresponding RefSeq identifications numbers for these proteins against the complete set of 859 proteins detected by mass spectrometry in this set of experiments.

### Supplemental Information

Additional information, including clinical data for all of the study participants, the complete list of proteins detected in each sample, percent peptide coverage for each protein and the expression levels for all genes in all samples are stored in a relational MYSQL database that is available at http://pulm.bumc.bu.edu/parce/parce.html. Microarray data from this study has been deposited in the National Center for Biotechnology Information Gene Expression Omnibus (GSE4635). Proteomic data has been deposited at Proteome Commons (http://www.proteomecommons.org/).

## Supporting Information

Figure S1 1D-PAGE of a current smoker sample prior to mass spectrometry. Proteins from each sample were separated by 1D-PAGE prior to mass spectrometry. A representative sample is shown. MW indicates the molecular weight marker. BSA indicates a bovine serum albumin standard. CS indicates current smoker.
(2.28 MB TIF)

Figure S2 1D-PAGE for approximation of protein yield prior to Western Blot. A small amount of material from each sample was retained for Western blotting. To roughly normalize the protein contribution from each sample, a small amount of material from the remaining samples were analyzed on 1D-PAGE and stained with Coomassie blue. MW indicates a molecular weight standard. NS indicates never smokers, and CS indicates current smokers.
(2.04 MB TIF)
