# Supplementary figures and images for: Comparison of Proteomic and Transcriptomic Profiles in the Bronchial Airway Epithelium of Current and Never Smokers
Source: PLoS One. 2009 Apr 9;4(4):e5043. doi: 10.1371/journal.pone.0005043 (PMC2664466; doi:10.1371/journal.pone.0005043)

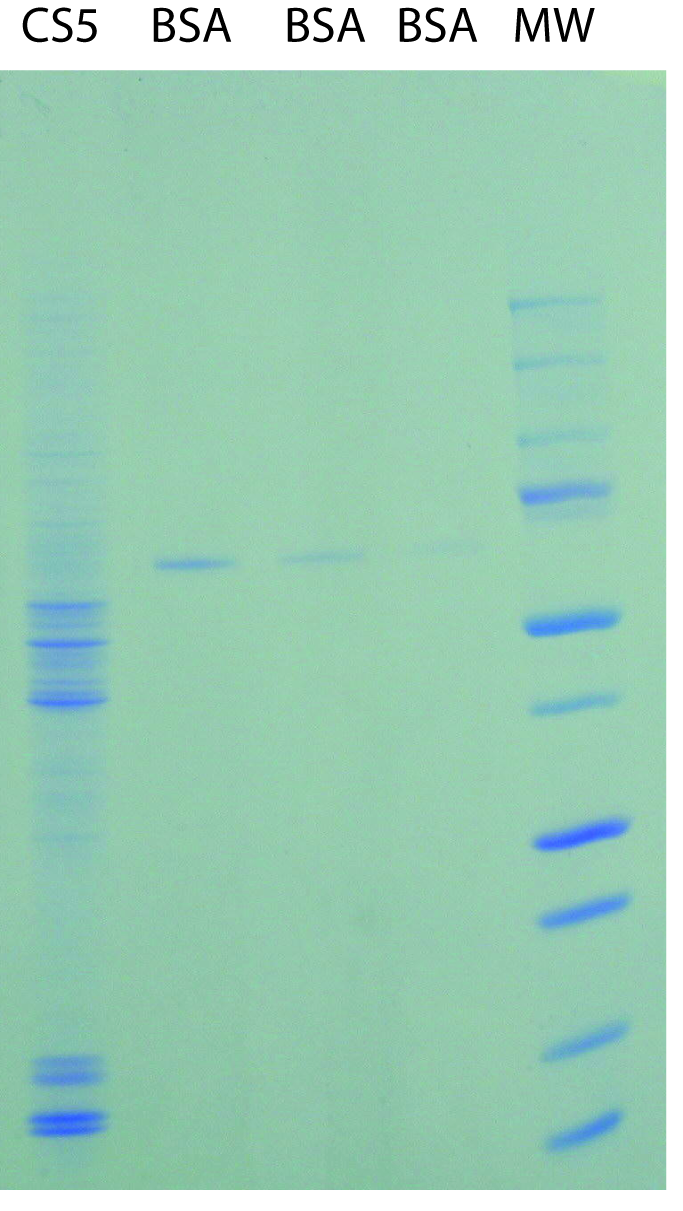

Supplement: Figure S1 — 1D-PAGE of a current smoker sample prior to mass spectrometry. Proteins from each sample were separated by 1D-PAGE prior to mass spectrometry. A representative sample is shown. MW indicates the molecular weight marker. BSA indicates a bovine serum albumin standard. CS indicates current smoker. (2.28 MB TIF) [file pone.0005043.s001.tif]

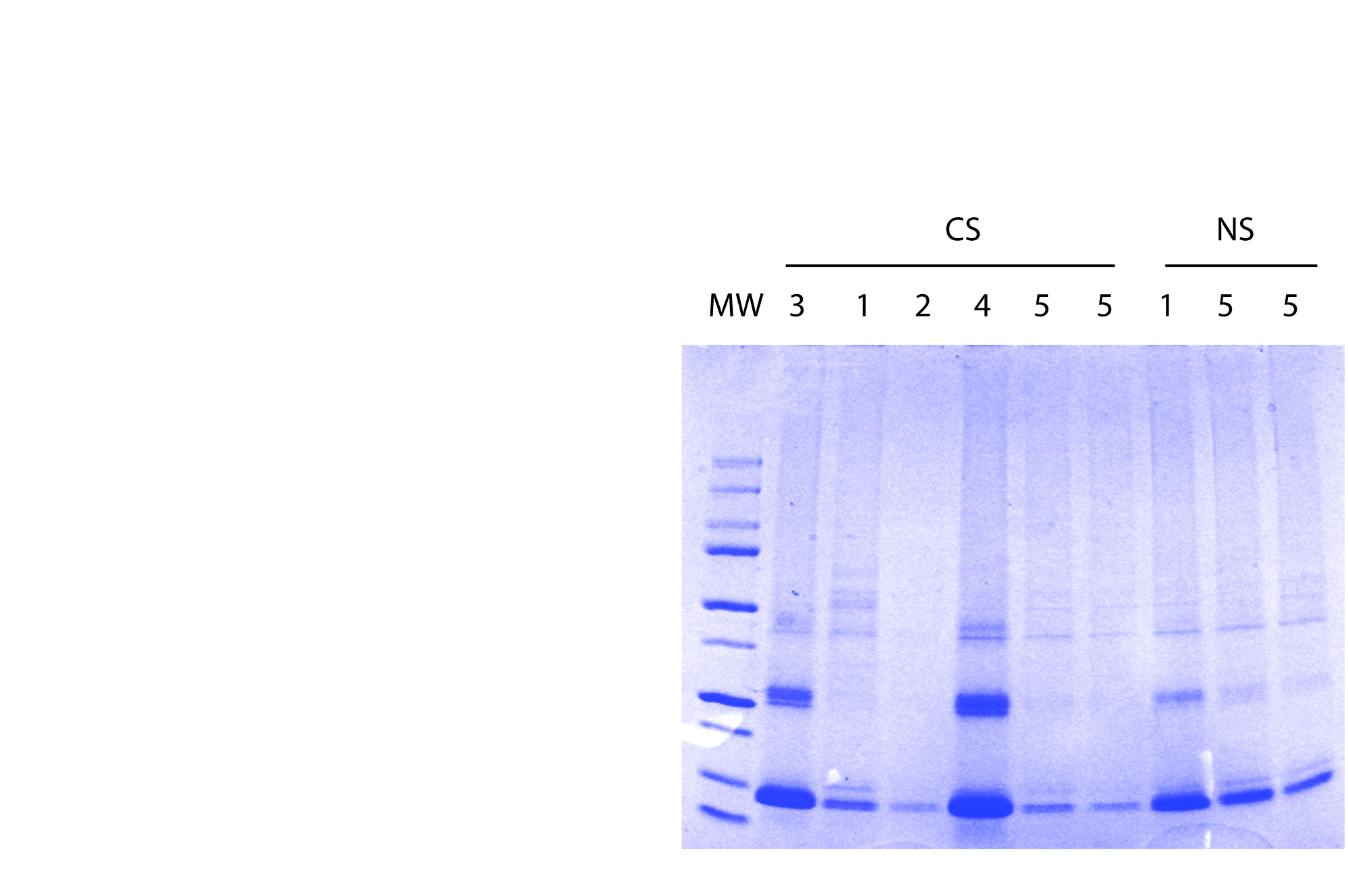

Supplement: Figure S2 — 1D-PAGE for approximation of protein yield prior to Western Blot. A small amount of material from each sample was retained for Western blotting. To roughly normalize the protein contribution from each sample, a small amount of material from the remaining samples were analyzed on 1D-PAGE and stained with Coomassie blue. MW indicates a molecular weight standard. NS indicates never smokers, and CS indicates current smokers. (2.04 MB TIF) [file pone.0005043.s002.tif]
